# Application of machine learning based on habitat imaging and vision transformer to predict treatment response of locally advanced esophageal squamous cell carcinoma following neoadjuvant chemoimmunotherapy: a multi-center study

**DOI:** 10.3389/fimmu.2025.1603249

**Published:** 2025-08-06

**Authors:** Shu-Han Xie, Hui Xu, Hai Zhang, Jin-Xin Xu, Shi-Jie Huang, Wen-Yi Liu, Zi-Lu Tang, Rong-Yu Xu, Sun-Kui Ke, Jin-Biao Xie, Qing-Yi Feng, Ming-Qiang Kang

**Affiliations:** ^1^ Department of Thoracic Surgery, Fujian Medical University Union Hospital, Fuzhou, Fujian, China; ^2^ The Graduate School of Fujian Medical University, Fuzhou, Fujian, China; ^3^ Department of Thoracic Surgery, Gaozhou People’s Hospital, Gaozhou, Guangdong,, China; ^4^ Department of Thoracic Surgery, Zhongshan Hospital of Xiamen University, School of Medicine, Xiamen University, Xiamen, China; ^5^ Department of Cardiothoracic Surgery, The Affiliated Hospital of Putian University, Putian, China; ^6^ Department of Thoracic Surgery, Cancer Hospital Chinese Academy of Medical Sciences, Shenzhen Center, Shenzhen, China; ^7^ Department of Thoracic Surgery, Quanzhou First Hospital, Quanzhou, Fujian,, China; ^8^ Department of Thoracic Surgery, Quanzhou First Hospital Affiliated to Fujian Medical University, Quanzhou, Fujian, China; ^9^ Department of Ultrasound, Gaozhou People’s Hospital, Gaozhou, Guangdong, China; ^10^ Key Laboratory of Cardio-Thoracic Surgery(Fujian Medical University), Fujian Province University, Fuzhou, Fujian, China; ^11^ Key Laboratory of Ministry of Education for Gastrointestinal Cancer, Fujian Medical University, Fuzhou, Fujian, China; ^12^ Fujian Key Laboratory of Tumor Microbiology, Fujian Medical University, Fuzhou, Fujian, China; ^13^ Clinical Research Center for Thoracic Tumors of Fujian Province, Fuzhou, Fujian, China

**Keywords:** neoadjuvant chemoimmunotherapy, treatment response, habitat imaging, vision transformer, machine learning, tumor subregions

## Abstract

**Objective:**

Current medical examinations and biomarkers struggle to assess the efficacy of chemoimmunotherapy (nICT) for locally advanced esophageal squamous cell carcinoma (ESCC). This study aimed to develop a machine learning model integrating habitat imaging and deep learning (DL) to predict the treatment response of ESCC patients to nICT.

**Methods:**

The study retrospectively collected 309 ESCC patients from 6 medical centers, divided into training and external validation cohorts. For habitat imaging analysis, intratumoral subregions were clustered using the K-means clustering method. DL features from intratumoral and peritumoral subregions were extracted by Vision Transformer (ViT) respectively and then subjected to feature selection. Subsequently, 11 machine learning models were constructed for predictive model. The model’s performance was evaluated using the area under the curve (AUC), decision curve analysis (DCA), calibration curve, and accuracy.

**Results:**

A total of 18 DL features were selected. The model of ExtraTrees, which was optimal, demonstrated superior performance with AUCs of 0.917 in training cohort and 0.831 in external validation cohort. Similarly, ExtraTrees showed good predictive capabilities in patients undergoing 2 cycles of nICT with AUC of 0.862 in validation cohort. This model also showed good calibration for prediction probability and satisfied clinical value on DCAs. Finally, the SHapley Additive exPlanations method elucidated the model’s precise predictions.

**Conclusion:**

The ExtraTrees model leveraging habitat imaging and ViT offered a non-invasive and accurate method to predict pathological response to nICT, guiding personalized treatment strategies, and decreasing the risk of immune-related adverse effects.

## 1 Introduction

Esophageal cancer (EC) is a prevalent malignant tumor in digestive system, ranking seventh in global incidence and sixth in mortality (1). Most EC patients are initially diagnosed with locally advanced esophageal squamous cell carcinoma (LA-ESCC). Neoadjuvant therapy followed by surgical resection is the standard treatment for these patients. In recent years, PD-1/PD-L1 inhibitors have shown promising results in treating ESCC. A meta-analysis suggested that neoadjuvant chemoimmunotherapy (nICT) elicited superior pathological responses than conventional neoadjuvant therapy (2). However, due to high degree of intratumoral heterogeneity and drug resistance, only 20%-40% of ESCC patients achieved pathological complete response (pCR) following nICT, and approximately 50% experienced major pathological response (MPR) (3–5). Besides, the use of nICT in ESCC patients is still in its early stage, facing multiple challenges, the most pressing of which is pinpointing those who are sensitive to nICT, given that immunotherapy comes with substantial medical expenses and poses a risk of severe immune-related adverse effects (irAEs).

Computed tomography (CT) and endoscopic ultrasound (EUS) are widely utilized to assess the efficacy of neoadjuvant therapy. However, evaluating the efficacy of neoadjuvant therapy with CT and EUS is challenging, mainly because of treatment-related factors like inflammation, edema, and fibrosis, as well as the distinct mechanisms of immunotherapy, which include delayed responses, pseudoprogression, hyperprogression, and mixed responses (6–9). Furthermore, these routine examinations, including PET-CT, fail to predict which patients can benefit from nICT prior to commencing neoadjuvant therapy. Additionally, the effects of immunotherapeutic drugs may manifest before notable changes in tumor size are observed (7). Hence, there is a pressing need for novel methods to predict and assess the efficacy of nICT in ESCC patients before treatment.

As a non-invasive technology, radiomics is extensively utilized in clinical decision-making (10). Radiomics assumes uniformity and homogeneity of the tumor within its volume of interest (VOI), analyzing the VOI in its entirety (11). However, imaging changes within the tumor region typically reflect distinct biological processes among tumoral subregions. Failing to consider these subregional variations may result in the oversight of subtle differences within the tumor, limiting the predictive power of imaging biomarkers. In contrast to radiomics, habitat imaging is a methodology that emphasizes tumor subregional analysis, enabling more effective quantification of tumor subregions associated with tumor growth and invasiveness, thus offering a more precise representation of tumor heterogeneity and drug sensitivity (12, 13). Recently, machine learning and deep learning have been extensively utilized in the medical field. Traditional machine learning predominantly relies on manually selected features (14). While, deep learning can automatically extract digital features from imaging data, potentially revealing new insights that were not previously recognized (15, 16). Therefore, this study aims to develop a machine learning model integrating habitat imaging and deep learning to predict the treatment response of ESCC patients to nICT.

## 2 Methods

### 2.1 Patient selection

This retrospective study included patients who underwent esophagectomy at 6 medical centers from August 1, 2019 to June 30, 2024. The inclusion criteria for this study were as follows (1): Histopathological confirmation of ESCC via endoscopic biopsy before treatment (2); Clinical stages of T_1-2_N_+_M_0_ or T_3-4a_N_any_M_0_ (3); Receipt of at least one cycle of nICT without restrictions on chemotherapy regimen or the type of immunodrug (3); Availability of CT images and complete clinicopathological data. The exclusion criteria for this study were: (1) Pathological diagnosis of non-squamous cell carcinoma; (2) Insufficient clinical information or pathological reports; (3) Poor-quality CT images or the presence of artifacts; (4) Refuse surgical intervention due to compromised cardiopulmonary function and other contributing factors. A total of 309 cases were ultimately included in this study.

Additionally, ESCC patients from our center (Fujian Medical University Union Hospital) were provided for a training cohort consisting of 198 individuals, while 111 patients from five other medical centers constituted an external validation cohort. The study is deemed to carry no risks to participants, and all data has been anonymized. The details of patient selection are summarized in **Supplementary Figure S1**.

### 2.2 Relevant definition and study endpoints

Pathological complete response (pCR) is defined as no residual tumor cells in both tumor tissue and lymph node. Major pathological response (MPR) is a condition wherein 10% or fewer viable tumor cells are within the resected primary esophageal tumor specimen. In this study, tumor regression grade (TRG) was utilized to assess the pathological response. In accordance with the guidelines established by the College of American Pathologists (CAP) and the National Comprehensive Cancer Network (NCCN), MPR was equated to TRG 0-1, while it corresponds to TRG 1–2 under the Mandard scoring system (17–19).

In this study, ESCC patients were classified into two groups: good-responder (GR) and poor-responder (PR). Notably, the GR group included MPR or higher, denoting complete or near-complete tumor regression. While, the PR group indicated cases with partial, minimal, or no tumor regression.

### 2.3 Treatment protocols

Under normal medical circumstances, diagnostic and clinical staging procedures included gastroscopy, contrast-enhanced computed tomography of the neck, chest, and upper abdomen, and neck ultrasound. PET-CT was performed when necessary.

In general, the specific medications utilized in nICT, including their formulations, subtly varied across different hospitals. The chemotherapy regimen primarily consisted of platinum in combination with paclitaxel or docetaxel, administered every three weeks. Common neoadjuvant chemotherapy regimens involved cisplatin (60 mg/m^2^) on day 1, followed by nab-paclitaxel (125 mg/m^2^) on days 1 and 8, or docetaxel (75 mg/m^2^) with cisplatin (60 mg/m^2^) on day 1. Following neoadjuvant chemotherapy, PD-1/PD-L1 inhibitors were administered. Generally, these inhibitors were also administered every three weeks, including sintilimab at a dosage of 200 mg, toripalimab at a dosage of 240 mg, pembrolizumab at a dosage of 200 mg, tislelizumab at a dosage of 200 mg and camrelizumab at a dosage of 200 mg.

The selection and adjustment of specific medications and their dosages were determined by expert oncologists and thoracic surgeons, considering drug-related toxicities and patient tolerance.

### 2.4 CT image acquisition and tumor segmentation

All ESCC patients in this study received comparable CT scans across six hospitals despite minor variations in the CT equipment and scanning protocols. The information on the scanning equipment and contrast agent injection protocols are provided in **Supplementary Material Part 1**. This study obtained venous phase CT images from the picture archiving and communication systems of six medical centers, utilizing the DICOM format for retrieval. The volume of interest (VOI) for the entire tumor is outlined by experienced radiologists. In general, areas where the esophageal wall thickness reaches or exceeds 5 millimeters are usually identified as regions of esophageal tumor lesions. Any disagreements that arose during segmentation were resolved through discussions. The 3D-Slicer software (version 4.11.20210226) was utilized in this process (20).

In order to minimize image heterogeneity, the pixel intensity was standardized by setting the window width to 350 and the window level to 40, and the images were resampled to achieve uniformity (1 mm * 1 mm * 1 mm).

### 2.5 intratumoral and peritumoral subregion generation

**Figure 1:**
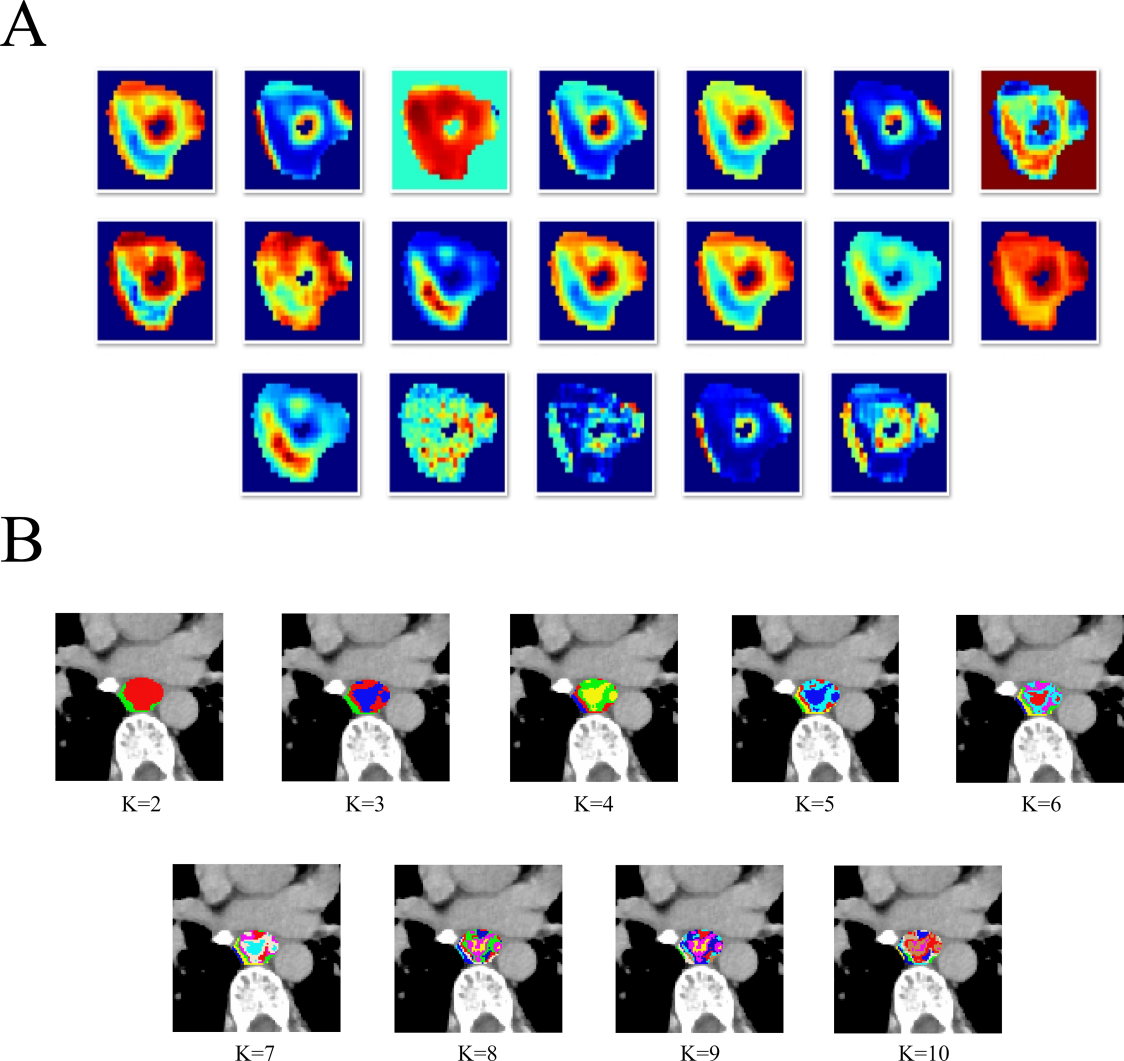
The heatmap of 19 CT-derived features **(A)**.The different tumor subregions based on different K-means values **(B)**.

During intratumoral subregions generation by habitat imaging, this study used 19 CT-derived features, including entropy, to cluster subregions. The details of these 19 CT-derived features can be found in **Supplementary Material Part 1** and **Figure 1A**. The K-means clustering ranging from 2 to 10 was performed to generate corresponding subregions, as is shown in **Figure 1B**. The optimal number of subregional divisions was determined using the Calinski-Harabasz index (CH index). Generally, a higher CH index indicates a more favorable clustering outcome. The details of K-means clustering and CH index were shown in **Supplementary Material Part 1**.

In this study, the optimal number of clusters, which was K=2, corresponds to the highest CH index, as is shown in **Supplementary Figure S2**. Therefore, the intratumoral region was divided into two heterogeneous subregions: subregion of Habitat 1 (H1) and subregion of Habitat 2 (H2). Besides, for peritumoral subregion generation, a 1-mm-wide band was generated with automated dilation of the tumor boundaries (peritumoral subregion). To focus on the VOI and reduce irrelevant background noise, these three subregions are respectively cropped out according to the outer cube around the edge of each tumor subregion, and subsequently utilized as input data for the following deep learning model construction.

### 2.6 3D Deep learning and quantitative feature extraction

During the training of the 3D deep learning model, the input images were resized to a standardized dimension of 64*64*48. The backbone network was constructed using Vision Transformer (ViT) architecture, and its generalization capability was enhanced via data augmentation techniques such as horizontal flipping, vertical flipping and random cropping. The transformer encoder included multi-head self-attention, multi-layer perceptron, residual connections and layer normalization. During the training phase, network parameters were updated through forward and backward propagation. Optimization was performed using the Adam optimizer with the cross-entropy loss function, and cosine annealing was used to dynamically adjust the learning rate. Additionally, the other hyperparameters of the model are as follows: the number of training epochs was set to 100, the batch size to 32, the initial learning rate to 0.001, and the dropout rate to 0.1. Finally, the weights of the trained Vision Transformer model were frozen and utilized as a deep learning (DL) feature extractor for subsequent tasks.

During the development of DL feature, sharing the same backbone architecture, each subregion was an independent input data and analyzed separately, thus forming an integrated model of three subregions. That is: the external cubes from each subregion were taken as input images, and deep learning was conducted respectively to extract DL features.

### 2.7 DL feature fusion and selection for subregional model

Initially, this study adopted feature-level fusion strategy, also known as early fusion, which involves concatenating DL features from three sub-regions into a single feature vector.

Then, the 1024 deep learning features from each of the three subregions were standardized using Z-scores to facilitate convergence. Two approaches were then employed to identify robust DL features. Initially, the correlations among highly repeatable features were evaluated using the Pearson correlation coefficient. When the correlation coefficient between any two features exceeded 0.9, one of the features was retained. Thereafter, the Least Absolute Shrinkage and Selection Operator (LASSO) analysis was utilized to screen out DL features and their corresponding coefficients that effectively predict poor-responder and good-responder groups.

### 2.8 Construction and evaluation of machine learning model of tumor subregions

After applying the LASSO feature selection method, 11 machine learning models were employed to integrate the selected features. These models encompassed logistic regression (LR), support vector machine (SVM), RandomForest (RF), GradientBoosting, ExtraTrees, AdaBoost, LightGBM, NaiveBayes, XGBoost, multilayer perceptron (MLP) and K Nearest Neighbors (KNN). The most optimal machine learning model was selected based on its performance in two cohorts, assessed by the area under the curve (AUC) and accuracy. The details of machine learning models and LASSO regression were shown in **Supplementary Material Part 1**.

**Figure 2:**
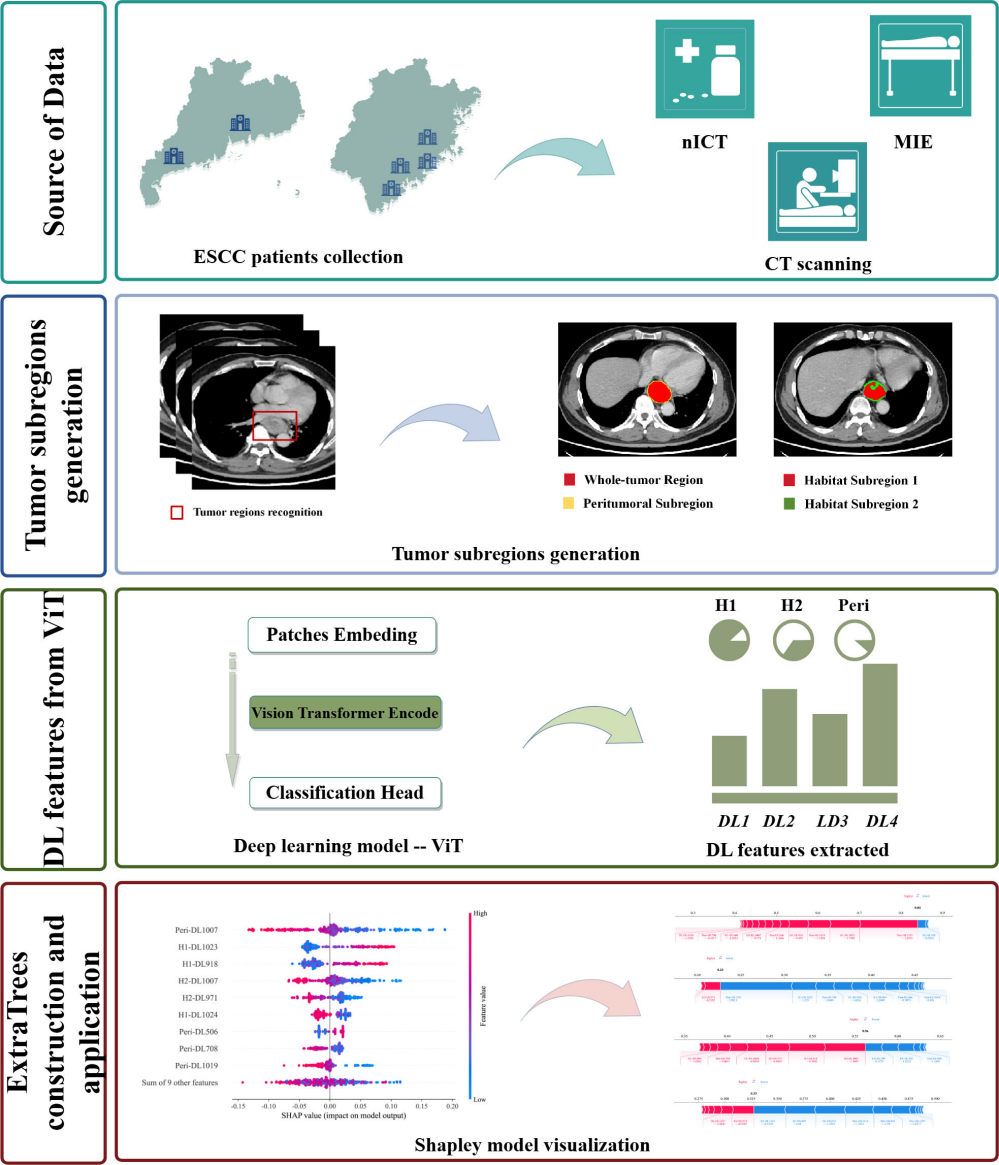
The flowchart of habitat imaging and deep learning analysis.

The diagnostic performance of the machine learning modes was assessed in two cohorts. Receiver Operating Characteristic (ROC) curves were generated to evaluate the diagnostic accuracy. Calibration performance was evaluated using calibration curves to determine their predictive reliability. Furthermore, decision curve analysis (DCA) was employed to assess the clinical utility of the optimal model. The SHapley Additive exPlanations (SHAP) method was used to elucidate the model’s prediction for each case. SHAP provides a reliable framework for accurately assessing the impact and contribution of each feature on the machine learning model. Moreover, each observation in the dataset can be interpreted based on its unique SHAP value. In addition, in subgroup analysis, the diagnostic performance of the optimal machine learning model was evaluated in subgroups of patients receiving different nICT cycles. The flowchart of habitat imaging and deep learning analysis is shown in **Figure 2**.

### 2.9 Statistical analysis

Continuous variables were described using median and interquartile range, and categorical variables were expressed by frequency and percentage. A two-tailed p-value of less than 0.05 was considered statistically significant. R software (version 4.0.2) and Python (version 3.7.12) were used for data analysis.

## 3 Results

### 3.1 Patient characteristics

A total of 309 ESCC patients were included in two cohorts (training cohort: 198 cases; external validation cohort: 111 cases). There were significant differences in the age (P = 0.002), smoking history (P < 0.001), treatment cycle (P = 0.004) and tumor location (P < 0.001) between two cohorts. Clinicopathological data of ESCC patients of two cohorts are detailed in **Table 1**.

**Table 1:** Clinicopathological characteristics of ESCC patients in training and external validation cohorts.

Variables	Training cohort	External validation cohort	P value
Sex			0.097
male	159 (80.30%)	80 (72.07%)	
female	39 (19.70%)	31 (27.93%)	
Age			0.002
≤65	148 (74.75%)	64 (57.66%)	
>65	50 (25.25%)	47 (42.34%)	
BMI			0.146
<18.5	25 (12.63%)	17 (15.32%)	
18.5-24	142 (71.72%)	68 (61.26%)	
≥24	31 (15.66%)	26 (23.42%)	
Smoking history			<0.001
negative	47 (23.74%)	77 (69.37%)	
positive	151 (76.26%)	34 (30.63%)	
Tumor location			<0.001
upper	15 (7.58%)	9 (8.11%)	
middle	92 (46.46%)	77 (69.37%)	
lower	91 (45.96%)	25 (22.52%)	
Treatment cycle			0.004
=2	114 (57.58%)	82 (73.87%)	
≠2	84 (42.42%)	29 (26.13%)	
Treatment response			0.297
PR	111 (56.06%)	69 (62.16%)	
GR	87 (43.94%)	42 (37.84%)	

PR, poor-responder; GR, good-responder.

### 3.2 Selection of DL features for machine learning model construction

Of the extracted DL features, 18 DL features with values of predicting PR and GR were obtained through LASSO regression analysis, as shown in **Supplementary Figure S3**. The coefficient values of the final selected features are shown in **Supplementary Figure S4**.

### 3.3 Selection of optimal machine learning model

**Figure 3:**
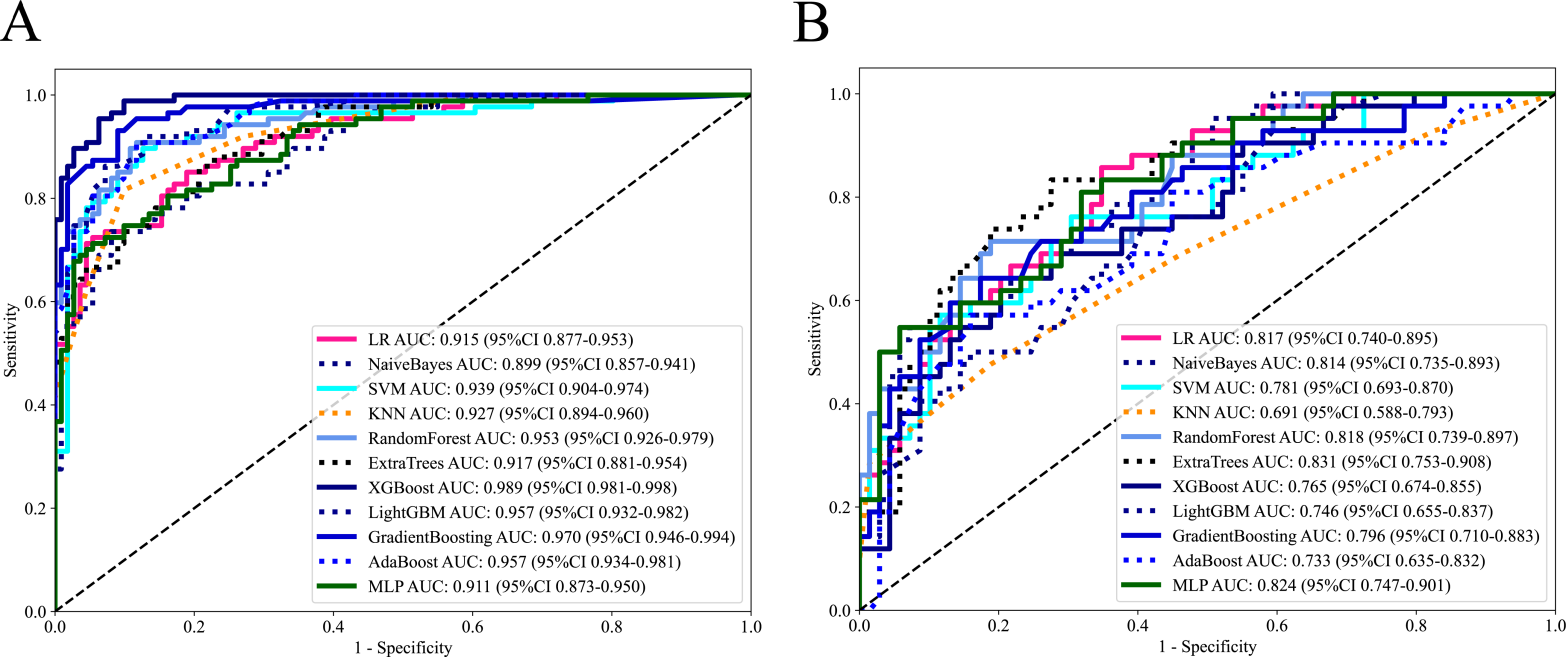
The ROC curves of each machine learning model in training cohort **(A)** and external validation cohort **(B)**.

A total of 11 machine learning models were constructed and evaluated to identify the optimal-performing model. All of the machine learning models used are shown in **Table 2**. The ExtraTrees model exhibited superior performance over other 10 models, achieving the highest AUC value while maintaining a well-balanced trade-off between sensitivity and specificity in two cohorts. The ROC analysis of each machine learning model in two cohorts is shown in **Figure 3**.

**Table 2:** The AUC, accuracy, sensitivity, and specificity of each machine learning model in terms of predicting treatment response.

Model name	AUC (95% CI)	Accuracy	Sensitivity	Specificity	Cohort
LR	0.915 (0.877 - 0.953)	0.848	0.724	0.946	training cohort
LR	0.817 (0.740 - 0.895)	0.730	0.857	0.652	validation cohort
NaiveBayes	0.899 (0.857 - 0.941)	0.838	0.736	0.919	training cohort
NaiveBayes	0.814 (0.735 - 0.893)	0.784	0.548	0.928	validation cohort
SVM	0.939 (0.904 - 0.974)	0.884	0.897	0.874	training cohort
SVM	0.781 (0.693 - 0.870)	0.721	0.762	0.696	validation cohort
KNN	0.927 (0.894 - 0.960)	0.864	0.816	0.901	training cohort
KNN	0.691 (0.588 - 0.793)	0.685	0.476	0.812	validation cohort
RandomForest	0.953 (0.926 - 0.980)	0.894	0.908	0.883	training cohort
RandomForest	0.818 (0.739 - 0.897)	0.775	0.714	0.812	validation cohort
ExtraTrees	0.917 (0.881 - 0.954)	0.823	0.862	0.793	training cohort
ExtraTrees	0.831 (0.753 - 0.908)	0.766	0.833	0.725	validation cohort
XGBoost	0.989 (0.981 - 0.998)	0.944	0.954	0.937	training cohort
XGBoost	0.765 (0.674 - 0.855)	0.73	0.619	0.797	validation cohort
LightGBM	0.957 (0.932 - 0.982)	0.894	0.920	0.874	training cohort
LightGBM	0.746 (0.655 - 0.837)	0.622	0.976	0.406	validation cohort
GradientBoosting	0.970 (0.946 - 0.994)	0.919	0.931	0.910	training cohort
GradientBoosting	0.796 (0.710 - 0.883)	0.757	0.643	0.826	validation cohort
AdaBoost	0.957 (0.934 - 0.981)	0.894	0.908	0.883	training cohort
AdaBoost	0.733 (0.635 - 0.832)	0.739	0.571	0.841	validation cohort
MLP	0.911 (0.873 - 0.950)	0.843	0.713	0.946	training cohort
MLP	0.824 (0.747 - 0.901)	0.730	0.810	0.681	validation cohort

AUC, area under curve; LR, Logistic Regression; SVM, Support Vector Machine; RF, RandomForest; KNN, K Nearest Neighbors; MLP, Multilayer Perceptron.

### 3.4 Performance evaluation of the ExtraTrees model

**Figure 4:**
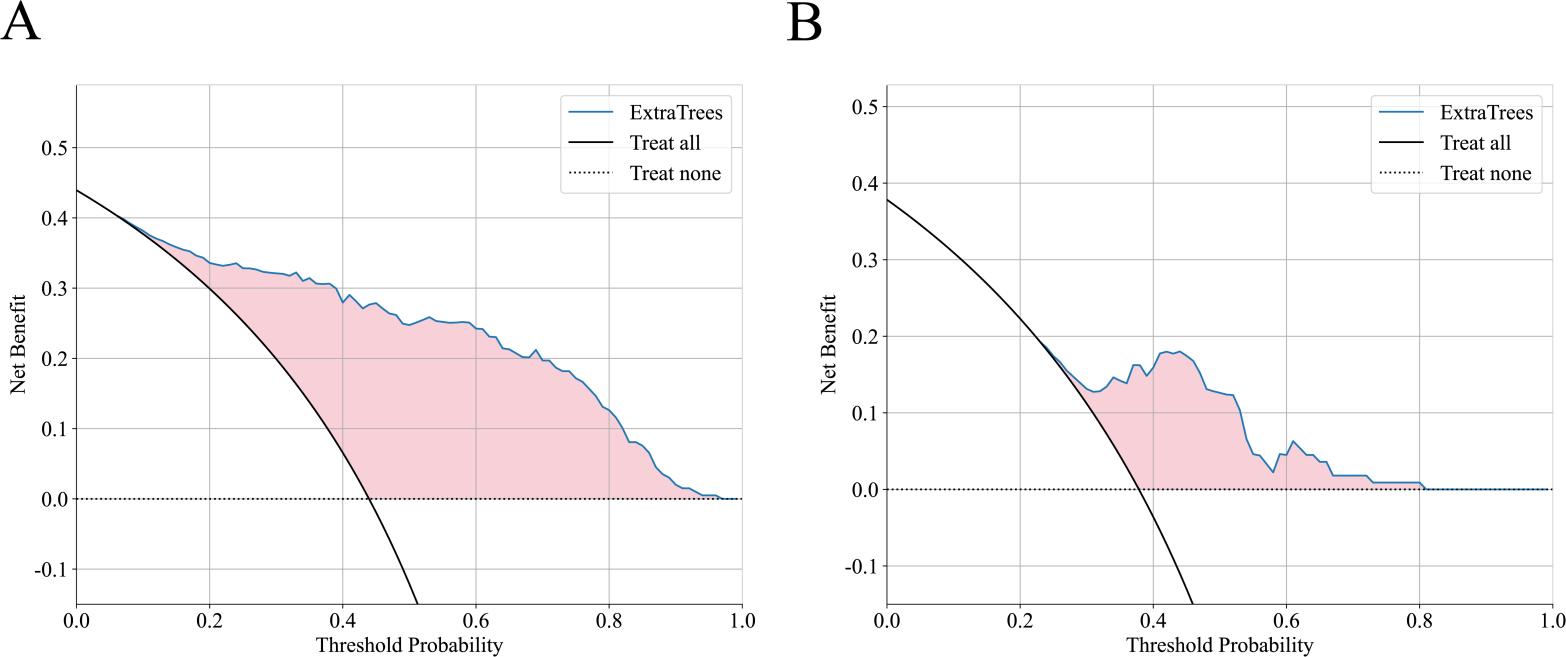
The decision curve analysis of ExtraTrees in training cohort **(A)** and external validation cohort **(B)**.

In this study, the DCAs for ExtraTrees were performed in training and external validation cohorts, as is shown in **Figure 4**. The decision curve analysis showed that the overall net benefit of ExtraTrees in the most reasonable threshold probability ranges (0.2 - 0.8), indicating the important clinical value in predicting treatment response. Calibration curves of ExtraTrees also showed good agreement between predicted and observed cases of PR and GR in two cohorts, as shown in **Supplementary Figure S5**.

### 3.5 The application of the ExtraTrees model in subgroup population

In two cohorts, the ExtraTrees model was utilized in patients undergoing different cycles of nICT (patients receiving 2 cycles and patients receiving other than 2 cycles). Notably, the model demonstrated good performance in the subgroup of patients receiving 2 treatment cycle with AUCs of 0.862 in validation cohort, thereby suggesting enhanced clinical versatility, as detailed in **Table 3**.

**Table 3:** The AUC, accuracy,sensitivity and specificity of ExtraTrees model in patients undergoing =2 cycles and ≠2 cycles of neoadjuvant therapy .

Treatment Cycle	AUC (95%CI)	Accuracy	Sensitivity	Specificity	Cohort
=2	0.933 (0.889 - 0.977)	0.842	0.860	0.828	training cohort
=2	0.862 (0.774 - 0.950)	0.780	0.857	0.741	validation cohort
≠2	0.896 (0.833 - 0.960)	0.798	0.865	0.745	training cohort
≠2	0.714(0.517 - 0.911)	0.724	0.786	0.667	validation cohort

AUC, area under curve.

### 3.6 Visualization of the ExtraTrees model

**Figure 5:**
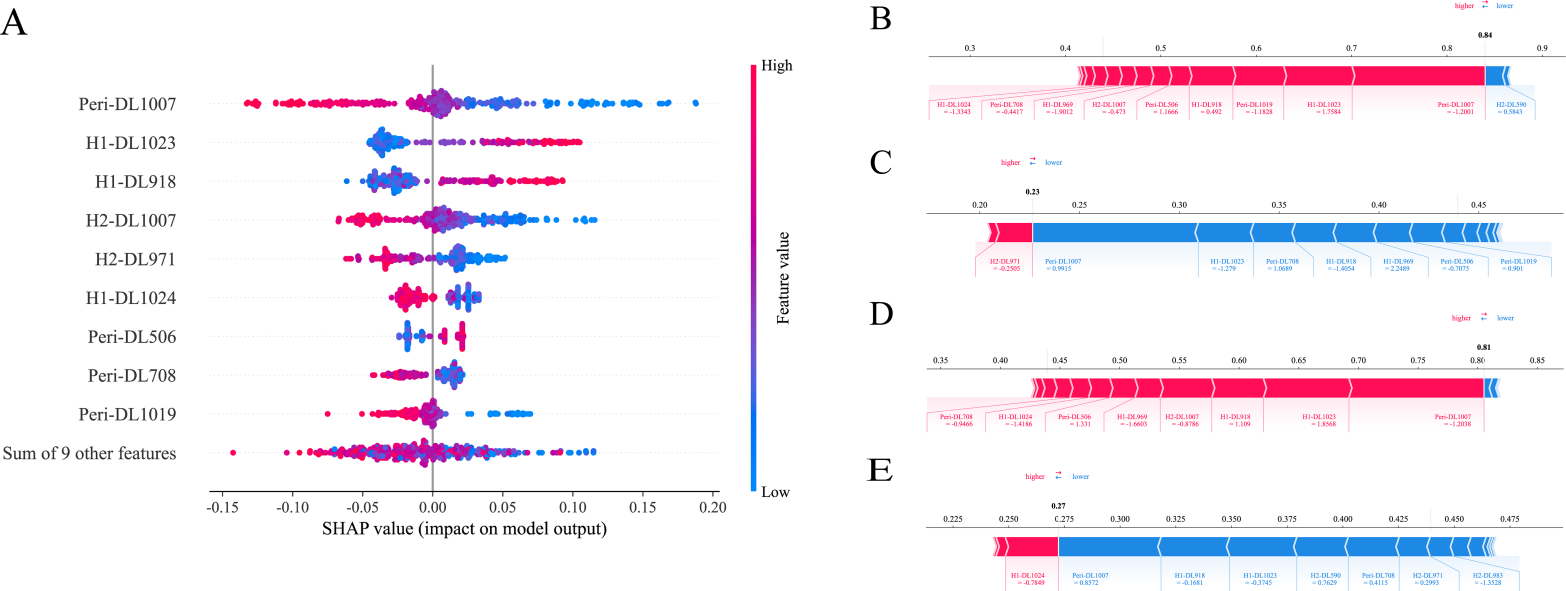
SHAP analysis of ExtraTrees model: The scatter plot of feature distributions using the SHAP analysis **(A)**. Force plot for patients with good treatment response in the training cohort **(B)** and external validation cohort **(D)**. Force plot for patients with poor treatment response in the training cohort **(C)** and external validation cohort **(E)**.

The SHAP method offers a framework that explains the outputs of the machine learning model of ExtraTrees and provides clear insights into the decision-making process for each case. The overall distribution of SHAP values for all selected features is illustrated in **Figure 5A**. In addition, the model’s performance was further elucidated by analyzing PR and GR cases in two cohorts: the two samples indicated that the model predicted achievement of GR, which transpired, as is shown in **Figures 5B, D**. While the other two cases demonstrated that the model predicted a failure to achieve GR, which indeed did not occur in each cohort, as is shown in **Figures 5C, E**.

## 4 Discussion

Despite advancements in screening and treatment regimens, the 5-year survival rate for LA-ESCC patients remains unsatisfied, primarily due to tumor heterogeneity and drug resistance (21, 22). Hence, predicting treatment responses and identifying potential beneficiary groups to nICT prior to the implementation of the treatment are vital for avoiding unnecessary adverse events and facilitating timely modifications to treatment protocols, thus improving the prognosis of ESCC.

MPR and pCR are the preferred indicators for assessing the treatment response to nICT (23). For ESCC patients attaining pCR, surgery or additional neoadjuvant treatment might not be requisite. However, aside from identifying which ESCC patients are capable of achieving pCR, another clinical challenge involves discerning those who can attain MPR, which also holds great guiding value and significance for clinical practice: Patients who do not achieve pCR after nICT do not necessarily demonstrate a poor treatment response, because some of these patients still achieve MPR, indicating good sensitivity to nICT. For patients unresponsive to nICT, surgery should be performed promptly or alternative curative treatments should be provided without delay in order to improve their therapeutic and survival outcomes (18). Furthermore, the additional rationale for selecting GR as the primary outcome of this study is as follows: Firstly, a previous study showed that the overall survival and recurrence-free survival of ESCC patients achieving MPR after nICT is significantly prolonged (24). In addition, while EC patients with microscopic residual disease are at a higher risk of recurrence, their survival rates are similar to those of patients with pCR (25). Similarly, previous study has also indicated that EC patients with a tumor regression response of ≥ 90% and no residual tumor cells in lymph nodes have survival outcomes similar to those with pCR (26). Therefore, this study categorized ESCC patients into good-responder and poor-responder groups.

In addition to common diagnostic examinations like CT or EUS, various biomarkers are used to assess the suitability of immunotherapy for EC patients, including PD-L1 expression level and tumor mutation burden (TMB). However, there is controversy over PD-L1’s ability to predict the treatment response of nICT in ESCC patients, given that various trials have shown that EC patients can benefit from the combination of immunotherapy and chemotherapy, irrespective of PD-L1 expression levels (27, 28). Moreover, certain studies have reported no significant difference in PD-L1 expression level between patients who exhibited good pathological responses and those who did not (3, 29). Likewise, TMB is a contentious predictor in the immunotherapy context (30). Moreover, these biomarkers are often extracted from a small portion of entire tumor samples through invasive, expensive, and time-consuming procedures. Given the high intratumoral heterogeneity, these biomarkers may not adequately reflect the entire spectrum of the tumor lesion’s characteristics. Therefore, in this study, the predictive model of ExtraTrees leveraging habitat imaging and ViT was constructed as non-invasive approach that can comprehensively capture the tumor characteristics. The ExtraTrees model achieved AUCs of 0.917 in the training cohort and 0.831 in the validation cohort, showing great potential of clinical application (preventing both premature discontinuation and unnecessary treatment extensions).

There is no consensus regarding the optimal number of cycles for nICT and the number of cycles for nICT in LA-ESCC varied across different medical centers (31, 32). Consequently, the ExtraTrees model was applied to subgroups of LA-ESCC patients receiving different numbers of nICT cycles, exhibiting satisfied predictive performance, thus highlighting its potential as a valuable tool for clinical decision-making. In this study, large proportion of ESCC patients received two cycles of nICT. With a larger sample size, the ExtraTrees model performed well in the subgroup of patients receiving 2 cycle of nICT. However, a smaller sample size of subgroup (patients receiving other than 2 cycle of nICT) may lead to insufficient statistical power and lower performance. Therefore, future study is needed to expand its sample size from more medical centers to enhance the reliability and representativeness of the analysis results.

In the field of medical oncology, predicting treatment response is a critical research focus. Compared to previous studies about treatment response predictions, our research offers several advantages: Firstly, this study established and externally validated the predictive model in a large sample size from 6 medical centers, confirming its reliability and stability. Secondly, the peritumoral features analyzed in this study serve as strong prognostic indicators, aligning with findings from other study (33). Evidences indicated that predictive models should consider the potential predictive capacity of the surrounding regions, capable of providing additional insights into tumor heterogeneity (34–39). Third, LA-ESCC exhibited high intratumoral heterogeneity across phenotypes, including proliferation, vascular distribution and oxygenation, which directly correlated with treatment resistance. Consequently, habitat imaging and clustering algorithms were utilized in this study to generate intratumoral subregions and then assess the tumor heterogeneity. In contrast to previous studies that analyzed the entire tumor region to predict the sensitivity to immunotherapy in ESCC patients (40, 41), our research focused on three tumor subregions for predicting treatment response of nICT. This novel approach not only provides a more accurate reflection of gene and transcriptome expression at the microscopic level but also facilitates a more effective analysis of the tumor micro-environment (the cell subpopulations within specific tumor regions) and tissue types (such as fibrosis and necrosis) at the macroscopic level. In addition, while Xie’s study on habitat analysis relied on clusters of Hounsfield Unit values and local entropy (42), our study employed 19 CT-derived features to generate tumor subregions, including Strength, RunVariance, DifferenceAverage, SmallAreaHighGrayLevelEmphasis and InverseVariance. These features have been proven to be strongly correlated with tumor aggressiveness and drug resistance (43–49), thereby allowing tumor region to be delineated into subregions from more imaging perspectives, providing a more nuanced reflection of intratumoral heterogeneity. Finally, the deep learning model of ViT utilized a self-attention mechanism to capture comprehensive image features without relying on adjacent element dependencies. Previous studies have shown that ViT outperformed Convolutional Neural Networks (CNNs) (50). Therefore, the ViT model was used as the feature extractor instead of CNNs, enhancing the efficiency of data processing and the capacity for generalization.

There were certain limitations in this study. First, the retrospective design inherently presents limitations despite strict patient selection to mitigate selection bias. Secondly, considerable efforts have been made to minimize variability in imaging data. However, discrepancies in CT equipment and protocols across different periods and institutions introduced bias and performance drop. Nonetheless, this variability is unavoidable and necessary, which could enhance the reproducibility and robustness of results from multi-institutional studies. Third, our study’s exclusive focus on ESCC patients may limit the model’s generalizability to esophageal adenocarcinoma. In addition, relevant data for investigating the biological mechanisms underlying treatment response predictions were limited due to the retrospective nature of this study. Future work will focus on systematically collecting such data and using multi-omics approaches to explore the relationship between biological processes and deep learning features. However, the substantial sample size in this study enhances the credibility of our findings.

## 5 Conclusion

In summary, by leveraging habitat imaging and vision transformer, the machine learning of ExtraTrees was constructed to enhance precision in predicting treatment response before initiating nICT, thereby avoiding unnecessary adverse events and facilitating timely modifications to treatment protocols. This machine learning model prevented premature discontinuation and unnecessary treatment extensions while relying on a comprehensive, non-invasive methodology. Future prospective studies will further validate the predictive performance of our findings in clinical practice.

## Data Availability

The raw data supporting the conclusions of this article will be made available by the authors, without undue reservation.
