# Supplementary material for: Application of machine learning based on habitat imaging and vision transformer to predict treatment response of locally advanced esophageal squamous cell carcinoma following neoadjuvant chemoimmunotherapy: a multi-center study
Source: Front Immunol. 2025 Aug 6;16:1603249. doi: 10.3389/fimmu.2025.1603249 (PMC12364654; doi:10.3389/fimmu.2025.1603249)
Supplement: Supplementary file 1 [file DataSheet1.docx]

**Supplementary Materials**

**Part 1**

**Supplementary Methods:**

**1 The information of scanner equipments and the scheme for intravenous contrast agent injection:**

All ESCC patients in this study received comparable CT scans across six hospitals, despite minor variations in the equipment and protocols utilized. Generally, patients are positioned for CT imaging according to the predefined scanning parameters set by the radiology department. The scan is performed with the patient in a supine position and in a calm, normal breathing state. The scan range extends from the level of the upper incisors to the lower border of the kidneys. The information of CT scanners in each medical center is presented below. Furthermore, to ensure uniformity, the reconstruction slice thickness and spacing between slices are both set at 2.5 mm.

| **Medical center** | **CT Scanner** | **Manufacturer** | **Tube Voltage** | **Tube Current** |
| --- | --- | --- | --- | --- |
| Fujian Medical University Union Hospital | Bright Speed Elite | GE | 120 kV | 220 mAs |
| Fujian Medical University Union Hospital | Discovery CT750 HD | GE | 120 kV | 50-220 mAs |
| Fujian Medical University Union Hospital | Somatom Definition | SIEMENS | 120 kV | 210 mAs |
| Quanzhou First Hospital | LightSpeed VCT | GE | 120 kV | 220 mAs |
| Quanzhou First Hospital | Optima CT680 Expert | GE | 120 kV | 220 mAs |
| Quanzhou First Hospital | Optima CT660 | GE | 120 kV | 220 mAs |
| Quanzhou First Hospital | SOMATOM Definition AS | SIEMENS | 120 kV | 220 mAs |
| Affiliated hospital of Putian university | Light Speed VCT | GE | 120 kV | 360 mAs |
| Affiliated hospital of Putian university | Somatom Definition Flash | SIEMENS | 120 kV | 360 mAs |
| Cancer Hospital Chinese Academy of Medical Sciences, Shenzhen Center | Revolution | GE | 120 kV | 250-500 mAs |
| Zhongshan Hospital Xiamen University | Iqon-Spectral | Philip | 120 kV | 73mAs |
| Zhongshan Hospital Xiamen University | Ingenuity | Philips | 120 kV | 101mAs |
| Zhongshan Hospital Xiamen University | Lightspeed VCT | GE | 120 kV | Auto |
| Zhongshan Hospital Xiamen University | Revolution | GE | 80-140 kV | Auto |
| Zhongshan Hospital Xiamen University | Somatom Definition Flash | Siemens | 100-120 kV | Auto |
| Zhongshan Hospital Xiamen University | Definition AS | Siemens | 120 kV | Auto |
| Gaozhou People's Hospital | Somatom Drive | Siemens | 120 kV | 130mAs |

For Fujian Medical University Union Hospital: After injecting a high-concentration iodinated contrast agent of 60 ml at a rate of 3 ml/s, a physiological saline flush at a rate of 3 ml/s is administered. The venous phase scan is performed 60 seconds after the start of contrast injection.

For Quanzhou First Hospital: After injecting iodine contrast agent of 1.2ml/Kg at a rate of 3.5 ml/s, a physiological saline flush at a rate of 3.5 ml/s is administered. The venous phase scan is performed 60 seconds after the start of contrast injection.

For Affiliated hospital of Putian university: After injecting iodine contrast agent of 1.2ml/Kg at a rate of 3.5 ml/s, a physiological saline flush at a rate of 2.5-3.0 ml/s is administered. The venous phase scan is performed 60 seconds after the start of contrast injection.

For Cancer Hospital Chinese Academy of Medical Sciences, Shenzhen Center: After injecting iodine contrast agent of 1.2ml/Kg, a physiological saline flush at a rate of 3.0 ml/s is administered. The venous phase scan is performed 50 seconds after the start of contrast injection.

For Zhongshan Hospital Xiamen University: After injecting iodine contrast agent of 60-70ml, a physiological saline flush at a rate of 3.2-3.5 ml/s is administered. The venous phase scan is performed 55-60 seconds after the start of contrast injection.

For Gaozhou People's Hospital: After injecting iodine contrast agent of 1.2ml/Kg, a physiological saline flush at a rate of 3.0 ml/s is administered. The venous phase scan is performed 50 seconds after the start of contrast injection.

**2 The 19 CT-derived features for clustering to generate intratumoral subregions (habitat imaging analysis)**

In this study, to divide the tumor region into distinct subregions from multiple imaging perspectives and provide a more precise characterization of intratumoral heterogeneity, 19 CT-derived features were selected for habitat imaging analysis. These 19 CT-derived features for clustering to generate habitat imaging in this study were:

1. **original_firstorder:** Entropy, MeanAbsoluteDeviation, Median;
2. **original_glcm:** DifferenceAverage, DifferenceEntropy, DifferenceVariance, Imc1, Imc2, InverseVariance, JointEnergy, JointEntropy, SumEntropy;
3. **original_glrlm:**SmallAreaHighGrayLevelEmphasis, RunVariance, SizeZoneNon UniformityNormalized, LongRunEmphasis, RunEntropy ;

4. **original_ngtdm:** Contrast, Strength.

These CT-derived features have been proven to be strongly correlated with tumor aggressiveness and drug resistance (1-7). Furthermore, these 19 CT-derived features are consistent with the features used in previous studies for habitat imaging(8-10). These features have been proven to be effective and feasible in the habitat imaging analysis and prediction task in these studies (8-10).

**3. The details of parameters in K-means algorithm, CH index, machine learning model and LASSO regression**

The K-means algorithm is a widely used clustering method, which has significant applications in image processing. In CT imaging, this algorithm can divide the volumes of interest in CT images into different subregions based on the gray value or CT-derived features. Specifically, for each pixel in the image, K-means determines its cluster by calculating the distance between it and each cluster center, and assigns it to the nearest cluster. Subsequently, the algorithm recalculates the positions of the cluster centers based on all the pixels currently belonging to the same cluster, that is, taking the average value of these pixels as the new center coordinates. In this study, number of clusters (K) was range from 2 to 10. The initialization method was chosen as k-means++. The n_init is set to 'auto'. For balancing accuracy and reducing computational cost, the tolerance was set as 0.0001. For controlling the running time of the algorithm and avoiding infinite loops, the number of maximum iterations was set as 300.

The optimal number of subregional divisions was identified using the Calinski-Harabasz index (CH index). This index is a commonly used metric for assessing the quality of clustering. This index represents the ratio of between-cluster variance to within-cluster variance, adjusted by the total number of clusters and data points. Specifically speaking, CH index evaluates both the separation between clusters and the cohesion within clusters, with higher values signifying more distinctly defined and well-separated clusters. A higher CH index indicates that the clusters are well-separated and compact, showing a more favorable clustering outcome. In this study, the optimal number of clusters, which was K=2, corresponded to the highest CH index, as is shown in Fig. S2.

In constructing the machine learning model, 11 algorithms were selected, including logistic regression, support vector machines, and random forests, to build predictive models. Besides, LASSO regression was used for feature selection. In the process of hyperparameter tunning, we adopted the default parameters provided by scikit-learn (can be found in [https://scikit-learn.org/stable/](https://scikit-learn.org/stable/)) and manually set to adjust the model's hyperparameters. For Random Forest (RF), the parameters were set as follows: n_estimators = 50, max_depth = 3, and min_samples_split = 4. For ExtraTrees, the parameters were configured as n_estimators = 80, max_depth = 3, and min_samples_split = 2. For LightGBM, the parameters were set to n_estimators = 20, max_depth = 4, and min_child_weight = 0.5. For XGBoost, the parameters were defined as n_estimators = 6, objective = 'binary:logistic', max_depth = 3, and min_child_weight = 0.2. For the LASSO regression, we employed 10-fold cross-validation to systematically evaluate 50 penalty coefficients ranging from 0.001 to 1, thereby determining the optimal penalty coefficient (λ).

**Reference**

1. Granata V, Fusco R, Setola SV, Brunese MC, Di Mauro A, Avallone A, et al. Machine learning and radiomics analysis by computed tomography in colorectal liver metastases patients for RAS mutational status prediction. *Radiol Med* (2024) 129(7):957-66. doi:10.1007/s11547-024-01828-5
2. Faggioni L, Gabelloni M, De Vietro F, Frey J, Mendola V, Cavallero D, et al. Usefulness of MRI-based radiomic features for distinguishing Warthin tumor from pleomorphic adenoma: performance assessment using T2-weighted and post-contrast T1-weighted MR images. *Eur J Radiol Open* (2022) 9:100429. doi:10.1016/j.ejro.2022.100429
3. Lucia F, Louis T, Cousin F, Bourbonne V, Visvikis D, Mievis C, et al. Multicentric development and evaluation of [(18)F]FDG PET/CT and CT radiomic models to predict regional and/or distant recurrence in early-stage non-small cell lung cancer treated by stereotactic body radiation therapy. *Eur J Nucl Med Mol Imaging* (2024) 51(4):1097-108. doi:10.1007/s00259-023-06510-y
4. Granata V, Fusco R, De Muzio F, Brunese MC, Setola SV, Ottaiano A, et al. Radiomics and machine learning analysis by computed tomography and magnetic resonance imaging in colorectal liver metastases prognostic assessment. *Radiol Med* (2023) 128(11):1310-32. doi:10.1007/s11547-023-01710-w
5. Jin N, Qiao B, Zhao M, Li L, Zhu L, Zang X, et al. Predicting cervical lymph node metastasis in OSCC based on computed tomography imaging genomics. *Cancer Med* (2023) 12(18):19260-71. doi:10.1002/cam4.6474
6. Wang Y, Feng G, Wang J, An P, Duan P, Hu Y, et al. Contrast-Enhanced Ultrasound-Magnetic Resonance Imaging Radiomics Based Model for Predicting the Biochemical Recurrence of Prostate Cancer: A Feasibility Study. *Comput Math Methods Med* (2022) 2022:8090529. doi:10.1155/2022/8090529
7. Li NY, Shi B, Chen YL, Wang PP, Wang CB, Chen Y, et al. The Value of MRI Findings Combined With Texture Analysis in the Differential Diagnosis of Primary Ovarian Granulosa Cell Tumors and Ovarian Thecoma-Fibrothecoma. *Front Oncol* (2021) 11:758036. doi:10.3389/fonc.2021.758036
8. Ye G, Wu G, Zhang C, Wang M, Liu H, Song E, et al. CT-based quantification of intratumoral heterogeneity for predicting pathologic complete response to neoadjuvant immunochemotherapy in non-small cell lung cancer. *Front Immunol* (2024) 15:1414954. doi:10.3389/fimmu.2024.1414954
9. Zhu Y, Zheng D, Xu S, Chen J, Wen L, Zhang Z, et al. Intratumoral habitat radiomics based on magnetic resonance imaging for preoperative prediction treatment response to neoadjuvant chemotherapy in nasopharyngeal carcinoma. *Jpn J Radiol* (2024) 42(12):1413-24. doi:10.1007/s11604-024-01639-8
10. Chen H, Liu Y, Zhao J, Jia X, Chai F, Peng Y, et al. Quantification of intratumoral heterogeneity using habitat-based MRI radiomics to identify HER2-positive, -low and -zero breast cancers: a multicenter study. *Breast Cancer Res* (2024) 26(1):160. doi:10.1186/s13058-024-01921-7

**Part 2**

**Supplementary Figure:**

**
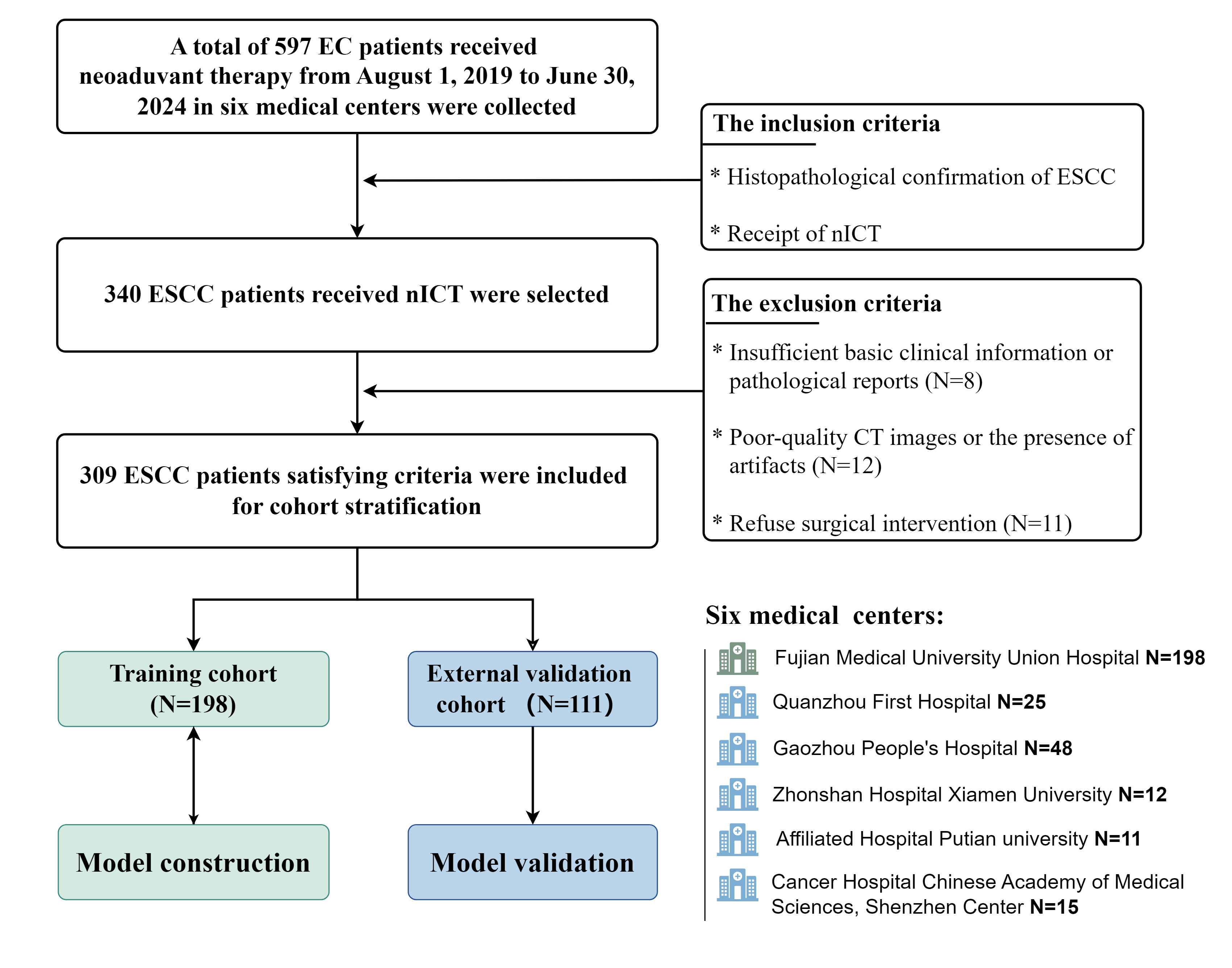
Fig. S1** the flow chart of the ESCC patient selection.

**
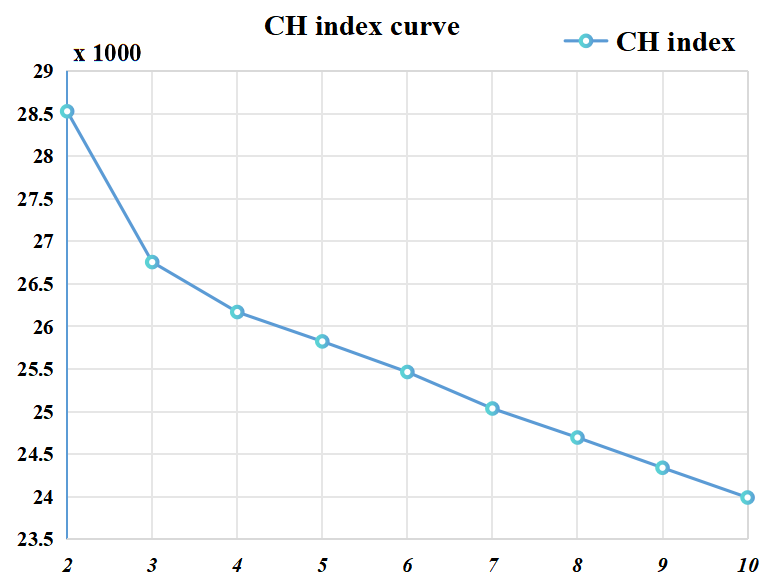
**

**Fig. S2** The line chart of Calinski-Harabasz index for cluster numbers.


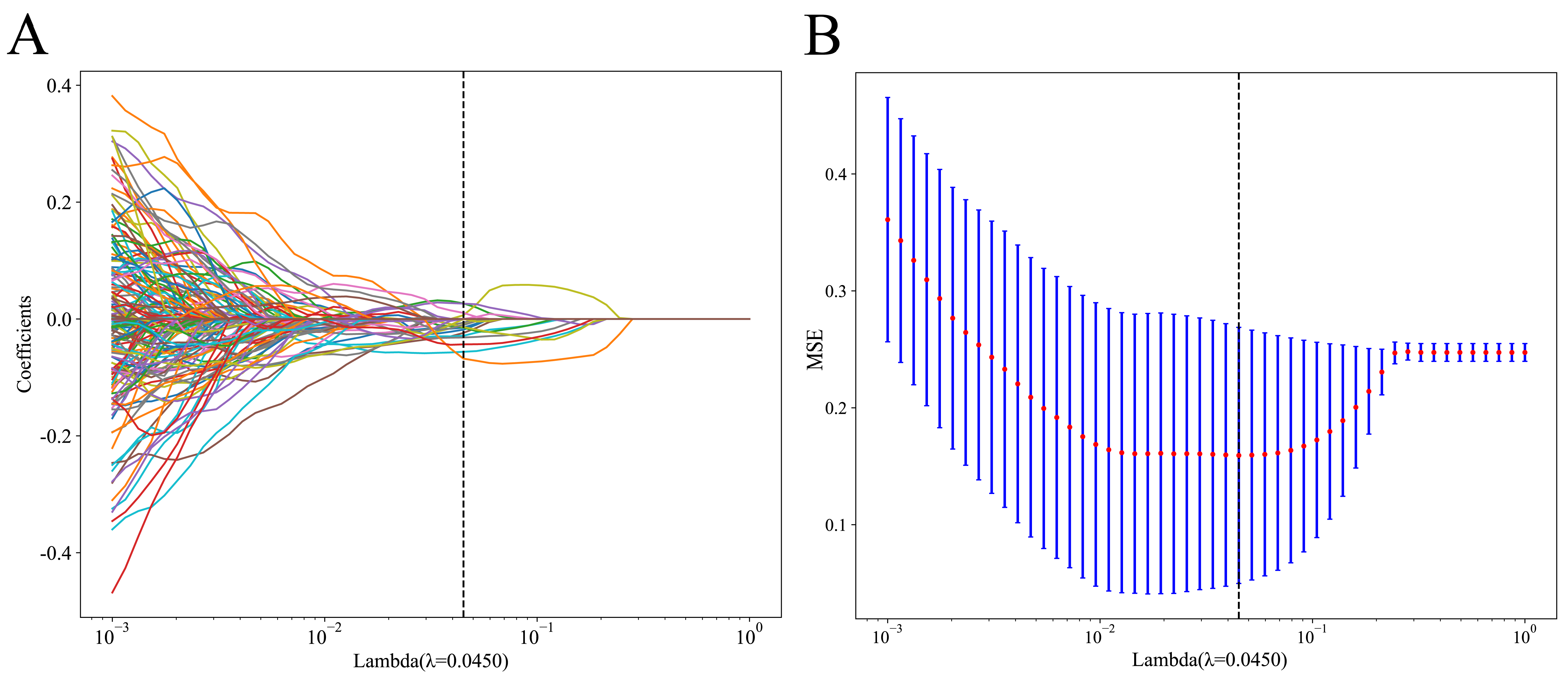


**Fig. S3** LASSO regression coefficient curve and Tuning parameter (λ) selection in Lasso model of radiomics features (A: coefficient curve; B: Tuning parameter (λ) selection in Lasso model)


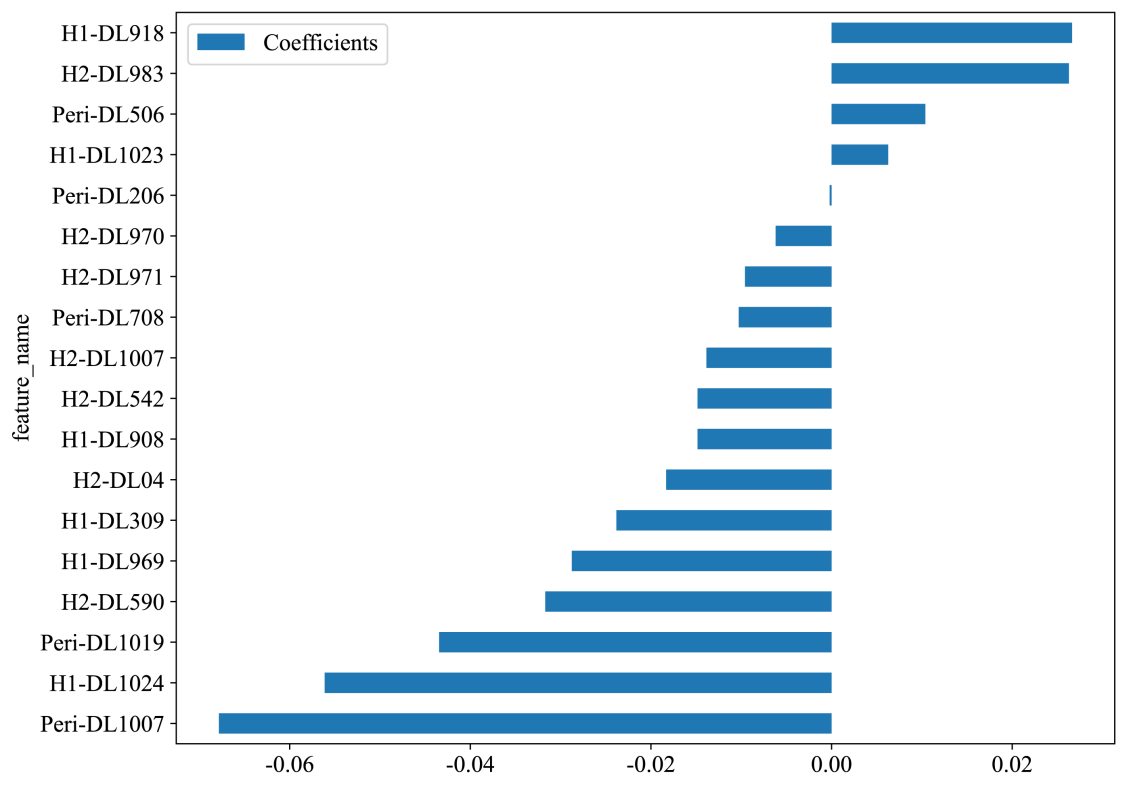


**Fig. S4** The histogram of coefficients of each selected feature.


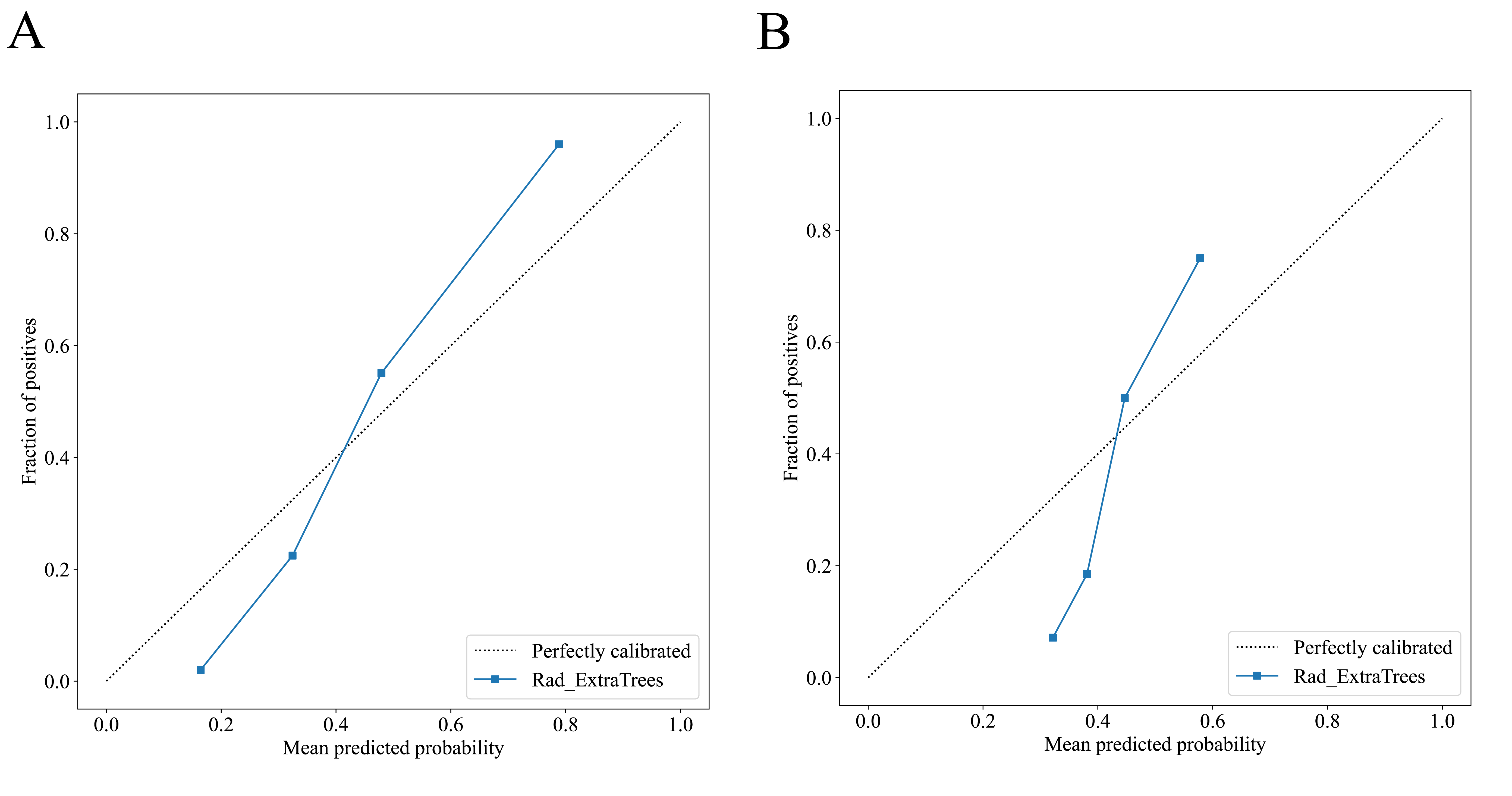


**Fig. S5** The calibration curve of ExtraTrees in training cohort (A) and external validation cohort (B).
